# (*Z*)-2-[(*E*)-2-(1-Benzothio­phen-3-yl­methyl­idene)hydrazin-1-yl­idene]-1,2-diphenyl­ethanone

**DOI:** 10.1107/S1600536812030978

**Published:** 2012-07-28

**Authors:** Merve Pekdemir, Şamil Işık, Sümeyye Gümüş, Erbil Ağar, Mustafa Serkan Soylu

**Affiliations:** a Department of Physics, Faculty of Arts and Sciences, Ondokuz Mayıs University, Kurupelit, TR-55139 Samsun, Turkey; b Department of Chemistry, Faculty of Arts and Sciences, Ondokuz Mayıs University, Kurupelit, TR-55139 Samsun, Turkey; c Department of Physics, Arts and Science Faculty, Giresun University, Giresun, Turkey

## Abstract

The title compound, C_23_H_16_N_2_OS, is not planar, the phenyl ring of the benzoyl group making a dihedral of 77.61 (7)° with the benzothio­phene system ring. The benzothio­phene system and the remaining phenyl ring make an angle of 12.71 (13)°. The conformation around the imine functions is *E* for the C=N bond towards the benzothio­phene system and *Z* for the C=N bond towards the benzoyl group. The packing of the mol­ecules shows C—H⋯π inter­actions. A weak intramolecular C—H⋯N bond also occurs.

## Related literature

For general background to benzothio­phenes, see: Katritzky *et al.* (1996 ▶); Shishoo & Jain (1992 ▶). For the biological properties of Schiff bases, see: Barton & Ollis (1979 ▶); Layer (1963 ▶); Ingold (1969 ▶). For industrial applications of Shiff bases, see: Taggi *et al.* (2002 ▶). For related structures, see: Dege *et al.* (2006 ▶, 2007 ▶); Demirtaş *et al.* (2009 ▶); Gül *et al.* (2007 ▶). For structural properties of benzothio­phene derivatives, see: Inamoto *et al.* (2008 ▶); Mlochowski & Potaczek (2009 ▶); Novopoltseva (1995 ▶). For reference bond-length data, see: Allen *et al.* (1987 ▶).
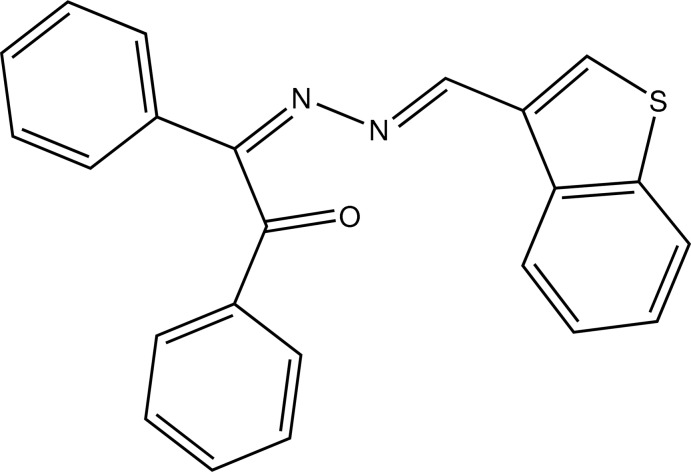


## Experimental

### 

#### Crystal data

C_23_H_16_N_2_OS
*M* *_r_* = 368.44
Monoclinic, 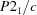

*a* = 17.1009 (7) Å
*b* = 8.7700 (4) Å
*c* = 13.1170 (6) Å
β = 103.898 (4)°
*V* = 1909.63 (15) Å^3^
*Z* = 4
Mo *K*α radiation
μ = 0.18 mm^−1^
*T* = 293 K
0.30 × 0.15 × 0.10 mm

#### Data collection

Oxford Diffraction SuperNova (single source at offset) Eos diffractometer
Absorption correction: multi-scan (*CrysAlis PRO*; Oxford Diffraction, 2007 ▶) *T*_min_ = 0.843, *T*_max_ = 1.000
7369 measured reflections
3815 independent reflections
2566 reflections with *I* > 2σ(*I*)
*R*_int_ = 0.025

#### Refinement

*R*[*F* ^2^ > 2σ(*F* ^2^)] = 0.054
*wR*(*F* ^2^) = 0.121
*S* = 1.07
3815 reflections
244 parameters
H-atom parameters constrained
Δρ_max_ = 0.20 e Å^−3^
Δρ_min_ = −0.24 e Å^−3^

### 

Data collection: *CrysAlis PRO* (Oxford Diffraction, 2007 ▶); cell refinement: *CrysAlis PRO*; data reduction: *CrysAlis PRO*; program(s) used to solve structure: *SHELXS97* (Sheldrick, 2008 ▶); program(s) used to refine structure: *SHELXL97* (Sheldrick, 2008 ▶); molecular graphics: *ORTEP-3 for Windows* (Farrugia, 1997 ▶); software used to prepare material for publication: *WinGX* (Farrugia, 1999 ▶).

## Supplementary Material

Crystal structure: contains datablock(s) I, global. DOI: 10.1107/S1600536812030978/vm2179sup1.cif

Structure factors: contains datablock(s) I. DOI: 10.1107/S1600536812030978/vm2179Isup2.hkl

Supplementary material file. DOI: 10.1107/S1600536812030978/vm2179Isup3.cml

Additional supplementary materials:  crystallographic information; 3D view; checkCIF report

## Figures and Tables

**Table 1:** Hydrogen-bond geometry (Å, °) *Cg*3 and *Cg*4 are the centroids of the C9–C14 and C18–C23 rings, respectively.

*D*—H⋯*A*	*D*—H	H⋯*A*	*D*⋯*A*	*D*—H⋯*A*
C20—H20⋯N2	0.93	2.53	3.083 (3)	118
C4—H4⋯*Cg*3^i^	0.93	2.74	3.648 (4)	167
C15—H15⋯*Cg*4^ii^	0.93	3.00	3.879 (3)	158

Symmetry codes: (i) 
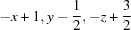
; (ii) 
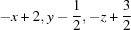
.

**Fig. 1.**
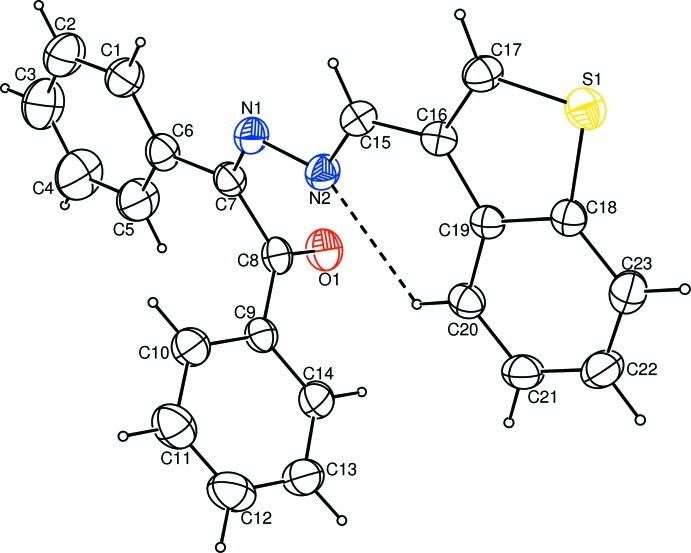
The molecular structure of the title compound, showing the atom-numbering scheme. Displacement ellipsoids are drawn at the 30% probability.

## supplementary crystallographic information

### Comment

Schiff bases, *i.e.*, compounds having a double C═N bond, are used as starting materials in the synthesis of important drugs, such as antibiotics and antiallergic, antiphlogistic and antitumor substances (Barton & Ollis, 1979; Layer, 1963; Ingold, 1969). On the industrial scale, they have a wide range of applications, such as dyes and pigments (Taggi *et al.*, 2002) and components of rubber compounds (Novopoltseva, 1995).

Benzothiophenes are significant heterocyles either as biological active or luminescent molecules (Shishoo & Jain, 1992; Katritzky *et al.*, 1996). Recently, new effective methods for the synthesis of benzothiophens have been developed (Inamoto *et al.*, 2008; Mlochowski & Potaczek, 2009). In this work, we report the crystal structure of a benzothiophene derivate containing C=N bonds.

The molecular structure is not planar (Fig.1), the dihedral angle between the C1—C6 benzene ring and the C9—C14 benzene ring is 89.59 (16)°. However, the dihedral angle between the C1—C6 benzene ring and the C18—C23 benzene ring is 12.62 (16)°. The thiophene ring is actually planar (maximum deviation 0.0132 (17) Å) and the dihedral angle between the benzene ring with thiophene ring is 1.05 (12)°. The benzothiophene ring system (C16—C23/S1) makes dihedral angles with the benzene rings of 12.71 (13)° for C1—C6 benzene ring and 77.62 (11)° for C9—C14 benzene ring.

The N═C double bond legths are 1.281 (3) Å for N1═C7 and 1.276 (3) Å for N2═C15. These are typical of double bonds, like to the matching bond length in (*E*)-2-[(3-trifluoromenthylphenylimino)methyl]-4-methylphenol [1.280 (2) Å; Gül *et al.*, 2007]. The C8═O1 bond length indicates the presence of a normal double C═O bond (Allen *et al.*, 1987). The C17—S1 and C19—S1 bond distances are 1.704 (2) Å and 1.733 (2) Å, respectively. The C—S bond distances are compatible with the literature (Dege *et al.*, 2006, 2007; Demirtaş *et al.*, 2009).

The molecules are packged by C—H···π and π···π interactions.

### Experimental

The compound (2*Z*)-2-[(2E)-(1-benzothiophen-3-ylmethylidene)hydrazinylidene] -1,2 diphenylethanone was prepared by refluxing a mixture of a solution containing 1-benzothiophene-3-carbaldehyde (0.012 g, 0.074 mmol) in 20 ml ethanol and a solution containing benzyl monohydrazone (0.017 g, 0.074 mmol) in 20 ml ethanol. The reaction mixture was stirred for 1 h under reflux. The crystals of (2*Z*)-2-[(2E)-(1-benzothiophen-3-ylmethylidene) hydrazinylidene]-1,2-diphenylethanone suitable for X-ray analysis were obtained from ethanol by slow evaporation (yield % 61; m.p 135–137 °C).

### Refinement

All H atoms were placed in calculated positions and constrained to ride on their parents atoms, with C—H = 0.93 Å and *U*_iso_(H)=1.2*U*_eq_(C).

### Crystal data

**Table:**

C_23_H_16_N_2_OS	*F*(000) = 768
*M**_r_* = 368.44	*D*_x_ = 1.282 Mg m^−^^3^
Monoclinic, *P*2_1_/*c*	Mo *K*α radiation, λ = 0.71073 Å
Hall symbol: -P 2ybc	Cell parameters from 2365 reflections
*a* = 17.1009 (7) Å	θ = 3.2–27.5°
*b* = 8.7700 (4) Å	µ = 0.18 mm^−^^1^
*c* = 13.1170 (6) Å	*T* = 293 K
β = 103.898 (4)°	Plate, yellow
*V* = 1909.63 (15) Å^3^	0.30 × 0.15 × 0.10 mm
*Z* = 4	

### Data collection

**Table:**

Oxford Diffraction SuperNova (single source at offset) Eos diffractometer	3815 independent reflections
Radiation source: fine-focus sealed tube	2566 reflections with *I* > 2σ(*I*)
Graphite monochromator	*R*_int_ = 0.025
Detector resolution: 16.0454 pixels mm^-1^	θ_max_ = 27.6°, θ_min_ = 3.2°
ω scans	*h* = −22→21
Absorption correction: multi-scan (*CrysAlis PRO*; Oxford Diffraction, 2007)	*k* = −10→6
*T*_min_ = 0.843, *T*_max_ = 1.000	*l* = −15→16
7369 measured reflections	

### Refinement

**Table:**

Refinement on *F*^2^	Primary atom site location: structure-invariant direct methods
Least-squares matrix: full	Secondary atom site location: difference Fourier map
*R*[*F*^2^ > 2σ(*F*^2^)] = 0.054	Hydrogen site location: inferred from neighbouring sites
*wR*(*F*^2^) = 0.121	H-atom parameters constrained
*S* = 1.07	*w* = 1/[σ^2^(*F*_o_^2^) + (0.0328*P*)^2^ + 0.4842*P*] where *P* = (*F*_o_^2^ + 2*F*_c_^2^)/3
3815 reflections	(Δ/σ)_max_ = 0.002
244 parameters	Δρ_max_ = 0.20 e Å^−^^3^
0 restraints	Δρ_min_ = −0.24 e Å^−^^3^

### Special details

**Table:**

Geometry. All e.s.d.'s (except the e.s.d. in the dihedral angle between two l.s. planes) are estimated using the full covariance matrix. The cell e.s.d.'s are taken into account individually in the estimation of e.s.d.'s in distances, angles and torsion angles; correlations between e.s.d.'s in cell parameters are only used when they are defined by crystal symmetry. An approximate (isotropic) treatment of cell e.s.d.'s is used for estimating e.s.d.'s involving l.s. planes.
Refinement. Refinement of *F*^2^ against ALL reflections. The weighted *R*-factor *wR* and goodness of fit *S* are based on *F*^2^, conventional *R*-factors *R* are based on *F*, with *F* set to zero for negative *F*^2^. The threshold expression of *F*^2^ > σ(*F*^2^) is used only for calculating *R*-factors(gt) *etc*. and is not relevant to the choice of reflections for refinement. *R*-factors based on *F*^2^ are statistically about twice as large as those based on *F*, and *R*- factors based on ALL data will be even larger.

### Fractional atomic coordinates and isotropic or equivalent isotropic displacement parameters (Å^2^)

**Table:**

	*x*	*y*	*z*	*U*_iso_*/*U*_eq_	
C1	0.63404 (17)	0.2840 (3)	0.5699 (2)	0.0696 (8)	
H1	0.6819	0.2657	0.5501	0.083*	
C2	0.5635 (2)	0.2214 (4)	0.5132 (3)	0.0925 (10)	
H2	0.5638	0.1615	0.4548	0.111*	
C3	0.4926 (2)	0.2463 (5)	0.5417 (3)	0.1115 (13)	
H3	0.4451	0.2028	0.5030	0.134*	
C4	0.49182 (19)	0.3354 (5)	0.6271 (3)	0.1178 (14)	
H4	0.4437	0.3526	0.6465	0.141*	
C5	0.56261 (17)	0.4000 (4)	0.6846 (3)	0.0901 (10)	
H5	0.5618	0.4610	0.7423	0.108*	
C6	0.63441 (15)	0.3744 (3)	0.6568 (2)	0.0588 (7)	
C7	0.71001 (14)	0.4422 (3)	0.71807 (18)	0.0516 (6)	
C8	0.71003 (13)	0.5224 (3)	0.82084 (19)	0.0522 (6)	
C9	0.70993 (13)	0.6910 (3)	0.82297 (19)	0.0507 (6)	
C10	0.69128 (16)	0.7746 (3)	0.7316 (2)	0.0640 (7)	
H10	0.6795	0.7249	0.6671	0.077*	
C11	0.68997 (18)	0.9321 (3)	0.7350 (3)	0.0817 (9)	
H11	0.6760	0.9881	0.6731	0.098*	
C12	0.70923 (18)	1.0052 (4)	0.8298 (3)	0.0875 (10)	
H12	0.7079	1.1112	0.8322	0.105*	
C13	0.73038 (17)	0.9241 (4)	0.9211 (3)	0.0811 (9)	
H13	0.7454	0.9747	0.9851	0.097*	
C14	0.72938 (15)	0.7666 (3)	0.9182 (2)	0.0657 (7)	
H14	0.7418	0.7113	0.9805	0.079*	
C15	0.90807 (14)	0.4568 (3)	0.73987 (18)	0.0508 (6)	
H15	0.9073	0.3914	0.6838	0.061*	
C16	0.98486 (13)	0.5071 (2)	0.80241 (17)	0.0453 (5)	
C17	1.05487 (14)	0.4515 (3)	0.78494 (18)	0.0541 (6)	
H17	1.0562	0.3823	0.7316	0.065*	
C18	1.08272 (13)	0.6305 (3)	0.93254 (17)	0.0460 (6)	
C19	1.00005 (13)	0.6127 (2)	0.88917 (16)	0.0413 (5)	
C20	0.94578 (15)	0.6956 (3)	0.93244 (17)	0.0506 (6)	
H20	0.8905	0.6849	0.9060	0.061*	
C21	0.97565 (17)	0.7928 (3)	1.01439 (19)	0.0620 (7)	
H21	0.9401	0.8490	1.0430	0.074*	
C22	1.05794 (19)	0.8089 (3)	1.0555 (2)	0.0688 (8)	
H22	1.0764	0.8758	1.1111	0.083*	
C23	1.11240 (17)	0.7285 (3)	1.01580 (19)	0.0601 (7)	
H23	1.1675	0.7391	1.0437	0.072*	
N1	0.77520 (12)	0.4280 (2)	0.68712 (15)	0.0569 (5)	
N2	0.84100 (12)	0.4976 (2)	0.75772 (15)	0.0533 (5)	
O1	0.70894 (11)	0.4453 (2)	0.89756 (14)	0.0710 (5)	
S1	1.13984 (4)	0.51911 (8)	0.86872 (5)	0.0595 (2)	

### Atomic displacement parameters (Å^2^)

**Table:**

	*U*^11^	*U*^22^	*U*^33^	*U*^12^	*U*^13^	*U*^23^
C1	0.0639 (18)	0.0671 (18)	0.0739 (18)	−0.0102 (15)	0.0091 (15)	−0.0083 (15)
C2	0.077 (2)	0.096 (2)	0.093 (2)	−0.015 (2)	−0.0014 (19)	−0.024 (2)
C3	0.063 (2)	0.132 (3)	0.122 (3)	−0.022 (2)	−0.013 (2)	−0.023 (3)
C4	0.0462 (19)	0.166 (4)	0.135 (3)	−0.015 (2)	0.011 (2)	−0.035 (3)
C5	0.0543 (18)	0.113 (3)	0.101 (2)	−0.0033 (18)	0.0145 (17)	−0.023 (2)
C6	0.0498 (15)	0.0572 (16)	0.0660 (16)	−0.0034 (13)	0.0075 (13)	0.0031 (14)
C7	0.0488 (14)	0.0463 (14)	0.0569 (15)	0.0009 (12)	0.0073 (12)	0.0034 (12)
C8	0.0380 (13)	0.0612 (16)	0.0553 (15)	0.0007 (12)	0.0070 (11)	0.0053 (13)
C9	0.0399 (13)	0.0557 (15)	0.0561 (14)	0.0034 (12)	0.0104 (11)	0.0000 (13)
C10	0.0716 (18)	0.0583 (17)	0.0625 (16)	−0.0013 (15)	0.0166 (14)	0.0042 (14)
C11	0.083 (2)	0.064 (2)	0.097 (2)	0.0007 (17)	0.0186 (18)	0.0164 (18)
C12	0.074 (2)	0.0573 (19)	0.127 (3)	−0.0005 (17)	0.015 (2)	−0.008 (2)
C13	0.069 (2)	0.075 (2)	0.092 (2)	0.0066 (17)	0.0054 (17)	−0.0267 (19)
C14	0.0563 (16)	0.075 (2)	0.0629 (17)	0.0112 (15)	0.0092 (13)	−0.0056 (15)
C15	0.0536 (15)	0.0504 (14)	0.0488 (13)	−0.0037 (12)	0.0131 (11)	−0.0048 (11)
C16	0.0461 (13)	0.0443 (13)	0.0479 (13)	−0.0011 (11)	0.0160 (11)	0.0011 (11)
C17	0.0550 (15)	0.0565 (15)	0.0537 (14)	−0.0022 (13)	0.0186 (12)	−0.0079 (12)
C18	0.0497 (14)	0.0420 (13)	0.0460 (13)	−0.0021 (11)	0.0109 (11)	0.0067 (11)
C19	0.0481 (13)	0.0365 (12)	0.0411 (12)	−0.0003 (11)	0.0143 (10)	0.0061 (10)
C20	0.0559 (15)	0.0472 (14)	0.0503 (14)	0.0045 (12)	0.0160 (12)	0.0057 (12)
C21	0.078 (2)	0.0545 (16)	0.0563 (16)	0.0100 (15)	0.0221 (14)	−0.0039 (13)
C22	0.091 (2)	0.0583 (17)	0.0535 (16)	−0.0025 (17)	0.0094 (15)	−0.0092 (13)
C23	0.0635 (17)	0.0570 (16)	0.0543 (15)	−0.0085 (14)	0.0034 (13)	0.0044 (13)
N1	0.0458 (12)	0.0620 (13)	0.0592 (13)	−0.0024 (11)	0.0055 (10)	−0.0073 (11)
N2	0.0465 (11)	0.0583 (13)	0.0533 (12)	−0.0032 (11)	0.0085 (9)	−0.0067 (10)
O1	0.0777 (13)	0.0735 (12)	0.0623 (11)	0.0000 (10)	0.0175 (10)	0.0162 (10)
S1	0.0460 (4)	0.0669 (5)	0.0675 (4)	0.0005 (3)	0.0172 (3)	−0.0010 (3)

### Geometric parameters (Å, º)

**Table:**

C1—C2	1.371 (4)	C12—H12	0.9300
C1—C6	1.387 (3)	C13—C14	1.381 (4)
C1—H1	0.9300	C13—H13	0.9300
C2—C3	1.369 (4)	C14—H14	0.9300
C2—H2	0.9300	C15—N2	1.276 (3)
C3—C4	1.369 (4)	C15—C16	1.440 (3)
C3—H3	0.9300	C15—H15	0.9300
C4—C5	1.384 (4)	C16—C17	1.362 (3)
C4—H4	0.9300	C16—C19	1.442 (3)
C5—C6	1.380 (4)	C17—S1	1.704 (2)
C5—H5	0.9300	C17—H17	0.9300
C6—C7	1.474 (3)	C18—C23	1.386 (3)
C7—N1	1.281 (3)	C18—C19	1.400 (3)
C7—C8	1.521 (3)	C18—S1	1.733 (2)
C8—O1	1.217 (3)	C19—C20	1.402 (3)
C8—C9	1.479 (3)	C20—C21	1.371 (3)
C9—C10	1.376 (3)	C20—H20	0.9300
C9—C14	1.383 (3)	C21—C22	1.388 (4)
C10—C11	1.382 (4)	C21—H21	0.9300
C10—H10	0.9300	C22—C23	1.367 (4)
C11—C12	1.367 (4)	C22—H22	0.9300
C11—H11	0.9300	C23—H23	0.9300
C12—C13	1.364 (4)	N1—N2	1.413 (3)
			
C2—C1—C6	120.3 (3)	C12—C13—C14	119.9 (3)
C2—C1—H1	119.8	C12—C13—H13	120.0
C6—C1—H1	119.8	C14—C13—H13	120.0
C3—C2—C1	120.6 (3)	C13—C14—C9	120.2 (3)
C3—C2—H2	119.7	C13—C14—H14	119.9
C1—C2—H2	119.7	C9—C14—H14	119.9
C2—C3—C4	119.9 (3)	N2—C15—C16	123.1 (2)
C2—C3—H3	120.1	N2—C15—H15	118.4
C4—C3—H3	120.1	C16—C15—H15	118.4
C3—C4—C5	120.0 (3)	C17—C16—C15	120.8 (2)
C3—C4—H4	120.0	C17—C16—C19	111.3 (2)
C5—C4—H4	120.0	C15—C16—C19	127.8 (2)
C6—C5—C4	120.4 (3)	C16—C17—S1	114.51 (18)
C6—C5—H5	119.8	C16—C17—H17	122.7
C4—C5—H5	119.8	S1—C17—H17	122.7
C5—C6—C1	118.7 (3)	C23—C18—C19	122.2 (2)
C5—C6—C7	120.7 (3)	C23—C18—S1	126.00 (19)
C1—C6—C7	120.6 (2)	C19—C18—S1	111.85 (17)
N1—C7—C6	120.3 (2)	C18—C19—C20	118.7 (2)
N1—C7—C8	120.7 (2)	C18—C19—C16	111.42 (19)
C6—C7—C8	118.9 (2)	C20—C19—C16	129.9 (2)
O1—C8—C9	122.7 (2)	C21—C20—C19	118.8 (2)
O1—C8—C7	118.6 (2)	C21—C20—H20	120.6
C9—C8—C7	118.7 (2)	C19—C20—H20	120.6
C10—C9—C14	119.2 (2)	C20—C21—C22	121.3 (2)
C10—C9—C8	121.2 (2)	C20—C21—H21	119.3
C14—C9—C8	119.7 (2)	C22—C21—H21	119.3
C9—C10—C11	120.4 (3)	C23—C22—C21	121.3 (2)
C9—C10—H10	119.8	C23—C22—H22	119.4
C11—C10—H10	119.8	C21—C22—H22	119.4
C12—C11—C10	119.8 (3)	C22—C23—C18	117.8 (2)
C12—C11—H11	120.1	C22—C23—H23	121.1
C10—C11—H11	120.1	C18—C23—H23	121.1
C13—C12—C11	120.5 (3)	C7—N1—N2	111.53 (19)
C13—C12—H12	119.7	C15—N2—N1	111.59 (19)
C11—C12—H12	119.7	C17—S1—C18	90.89 (11)
			
C6—C1—C2—C3	−0.4 (5)	C8—C9—C14—C13	179.3 (2)
C1—C2—C3—C4	0.5 (6)	N2—C15—C16—C17	175.2 (2)
C2—C3—C4—C5	−0.1 (6)	N2—C15—C16—C19	−3.0 (4)
C3—C4—C5—C6	−0.4 (6)	C15—C16—C17—S1	−178.11 (17)
C4—C5—C6—C1	0.5 (5)	C19—C16—C17—S1	0.3 (3)
C4—C5—C6—C7	−179.7 (3)	C23—C18—C19—C20	−0.7 (3)
C2—C1—C6—C5	−0.1 (4)	S1—C18—C19—C20	180.00 (15)
C2—C1—C6—C7	−179.9 (3)	C23—C18—C19—C16	178.8 (2)
C5—C6—C7—N1	−174.5 (3)	S1—C18—C19—C16	−0.4 (2)
C1—C6—C7—N1	5.3 (4)	C17—C16—C19—C18	0.1 (3)
C5—C6—C7—C8	8.4 (4)	C15—C16—C19—C18	178.4 (2)
C1—C6—C7—C8	−171.8 (2)	C17—C16—C19—C20	179.6 (2)
N1—C7—C8—O1	−101.5 (3)	C15—C16—C19—C20	−2.1 (4)
C6—C7—C8—O1	75.6 (3)	C18—C19—C20—C21	1.0 (3)
N1—C7—C8—C9	79.5 (3)	C16—C19—C20—C21	−178.4 (2)
C6—C7—C8—C9	−103.3 (3)	C19—C20—C21—C22	−0.7 (4)
O1—C8—C9—C10	−163.7 (2)	C20—C21—C22—C23	0.0 (4)
C7—C8—C9—C10	15.1 (3)	C21—C22—C23—C18	0.4 (4)
O1—C8—C9—C14	16.7 (3)	C19—C18—C23—C22	0.0 (3)
C7—C8—C9—C14	−164.4 (2)	S1—C18—C23—C22	179.19 (19)
C14—C9—C10—C11	−1.7 (4)	C6—C7—N1—N2	−178.57 (19)
C8—C9—C10—C11	178.8 (2)	C8—C7—N1—N2	−1.5 (3)
C9—C10—C11—C12	1.6 (4)	C16—C15—N2—N1	−178.0 (2)
C10—C11—C12—C13	0.5 (5)	C7—N1—N2—C15	166.1 (2)
C11—C12—C13—C14	−2.5 (5)	C16—C17—S1—C18	−0.51 (19)
C12—C13—C14—C9	2.4 (4)	C23—C18—S1—C17	−178.7 (2)
C10—C9—C14—C13	−0.3 (4)	C19—C18—S1—C17	0.54 (17)

### Hydrogen-bond geometry (Å, º)

Cg3 and Cg4 are the centroids of the C9–C14 and C18–C23 rings, respectively.

**Table:**

*D*—H···*A*	*D*—H	H···*A*	*D*···*A*	*D*—H···*A*
C20—H20···N2	0.93	2.53	3.083 (3)	118
C4—H4···*Cg*3^i^	0.93	2.74	3.648 (4)	167
C15—H15···*Cg*4^ii^	0.93	3.00	3.879 (3)	158

Symmetry codes: (i) −*x*+1, *y*−1/2, −*z*+3/2; (ii) −*x*+2, *y*−1/2, −*z*+3/2.
